# Association of overweight with treatment outcomes in pulmonary tuberculosis

**DOI:** 10.1186/s12879-025-11669-w

**Published:** 2025-10-08

**Authors:** Kyung Hoon Kim, Hyung Woo Kim, Eung Gu Lee, Yeonhee Park, Sung Soo Jung, Jin Woo Kim, Jee Youn Oh, Heayon Lee, Seung Hoon Kim, Sun-Hyung Kim, Jiwon Lyu, Yousang Ko, Sun Jung Kwon, Yun-Jeong Jeong, Do Jin Kim, Hyeon-Kyoung Koo, Ganghee Chae, Sun Young Kyung, Sung Soon Lee, Jae Seuk Park, Yoolwon Jeong, Ju Sang Kim, Jinsoo Min

**Affiliations:** 1 Division of Pulmonary and Critical Care Medicine, Department of Internal Medicine, Incheon St. Mary’s Hospital, College of Medicine, The Catholic University of Korea, Seoul, Republic of Korea; 2 Division of Pulmonary, Allergy and Critical Care Medicine, Department of Internal Medicine, Bucheon St. Mary’s Hospital, College of Medicine, The Catholic University of Korea, Seoul, Republic of Korea; 3 Division of Pulmonary and Critical Care Medicine, Department of Internal Medicine, Daejeon St. Mary’s Hospital, College of Medicine, The Catholic University of Korea, Seoul, Republic of Korea; 4 Division of Pulmonary and Critical Care Medicine, Department of Internal Medicine, Chungnam National University Hospital, Daejeon, Republic of Korea; 5 Division of Pulmonary and Critical Care Medicine, Department of Internal Medicine, College of Medicine, Uijeongbu St. Mary’s Hospital, The Catholic University of Korea, Seoul, Republic of Korea; 6 Division of Pulmonary, Allergy, and Critical Care Medicine, Department of Internal Medicine, Korea University Guro Hospital, Korea University College of Medicine, Seoul, Republic of Korea; 7 Division of Pulmonary, Critical Care and Sleep Medicine, Department of Internal Medicine, Eunpyeong St. Mary’s Hospital, College of Medicine, The Catholic University of Korea, Seoul, Republic of Korea; 8 Division of Pulmonology, Department of Internal Medicine, College of Medicine, St. Vincent’s Hospital, The Catholic University of Korea, Seoul, Republic of Korea; 9 Division of Pulmonary and Critical Care Medicine, Department of Internal Medicine, Chungbuk National University Hospital, Cheongju, Republic of Korea; 10 Department of Pulmonary and Critical Care Medicine, Soonchunhyang University Cheonan Hospital, Soonchunhyang University College of Medicine, Cheonan, Republic of Korea; 11 Division of Pulmonary, Allergy and Critical Care Medicine, Department of Internal Medicine, Kangdong Sacred Heart Hospital, Hallym University College of Medicine, Seoul, Republic of Korea; 12 Division of Pulmonary and Critical Care Medicine, Department of Internal Medicine, Konyang University Hospital, Konyang University College of Medicine, Daejeon, Korea; 13 Division of Pulmonary and Critical Care Medicine, Department of Internal Medicine, Dongguk University Ilsan Hospital, Goyang, Republic of Korea; 14 Division of Allergy and Respiratory Medicine, Soonchunhyang University Bucheon Hospital, Soonchunhyang University College of Medicine, Bucheon, Republic of Korea; 15 Division of Pulmonary and Critical Care Medicine, Department of Internal Medicine, Ilsan Paik Hospital, Inje University College of Medicine, Goyang, Republic of Korea; 16 Division of Pulmonary and Critical Care Medicine, Department of Internal Medicine, Ulsan University Hospital, Ulsan University College of Medicine, Ulsan, Republic of Korea; 17 Division of Pulmonology, Departments of Internal Medicine, Gachon University Gil Hospital, Incheon, Republic of Korea; 18 Division of Pulmonology, Department of Internal Medicine, Dankook University College of Medicine, Cheonan, Republic of Korea; 19 Department of Preventive Medicine, Dankook Universy Hospital, Dankook University College of Medicine, Cheonan, Republic of Korea; 20 Division of Pulmonary and Critical Care Medicine, Department of Internal Medicine, Seoul St. Mary’s Hospital, College of Medicine, The Catholic University of Korea, 222 Banpo-daero, Seocho-gu, Seoul, 06591 Republic of Korea

**Keywords:** Tuberculosis, Diabetes, Body mass index, Overweight, Treatment outcome

## Abstract

**Background:**

While overweight has been associated with a reduced risk of developing tuberculosis and diabetes with an increased risk, it remains unclear how these conditions influence anti-tuberculosis treatment outcomes. We aimed to examine the association of overweight with anti-tuberculosis treatment outcomes, and to evaluate whether this association differs by diabetes status, using two Korean cohorts.

**Methods:**

Among patients with pulmonary tuberculosis enrolled in the multicenter prospective cohort study of pulmonary tuberculosis (COSMOTB) and the Korea Tuberculosis Cohort (KTBC) registry, we defined overweight as BMI ≥ 23 kg/m² according to national criteria and compared it with normal/underweight (BMI < 23 kg/m², per criteria). The primary and secondary outcomes were unfavorable outcomes and mortality. Multivariable regression analysis was conducted to evaluate the association of overweight with treatment outcomes, adjusting for potential confounders. Subgroup analyses were performed to assess the association in patients with and without diabetes.

**Results:**

In the COSMOTB dataset, the proportion of overweight individuals was 34.4%. Overweight was associated with a lower odds of unfavorable treatment outcome (adjusted odds ratio [aOR], 0.61; 95% confidence interval [CI], 0.37–0.97) and all-cause mortality during treatment (aOR, 0.49; 95% CI, 0.24–0.93). In subgroup analyses, these associations were observed in patients with diabetes but not in those without diabetes. In the KTBC database, overweight was also associated with reduced odds of unfavorable outcome in patients with diabetes.

**Conclusion:**

In this observational study, overweight was associated with improved treatment outcomes in pulmonary TB. This association was also significantly observed in patients with diabetes; however, causality cannot be inferred.

**Supplementary Information:**

The online version contains supplementary material available at 10.1186/s12879-025-11669-w.

## Introduction

Tuberculosis (TB) remains a significant global health burden even after the end of the coronavirus disease 2019 pandemic [1]. This is particularly true in the low-income countries where TB-related mortality rates remain high. Similarly, obesity and diabetes have become major global health issues, with their prevalence increasing post-pandemic [2–4]. The complications and mortality rates associated with these conditions have risen, especially in the high-income countries.

Obesity and diabetes are closely interlinked. Numerous studies have demonstrated that higher body mass index (BMI) increases the likelihood of developing diabetes [5, 6]. Weight loss is the primary treatment for individuals with prediabetes [7]. However, the impact of these two conditions on TB is not well understood. A high BMI is known to be a protective factor against TB, while a low BMI, or underweight, is a known risk factor for both the incidence of TB and TB-related mortality [8]. Diabetes also increases the risk of developing TB and negatively affects treatment success rates [9–11]. Obesity raises the risk of diabetes but offers protection against TB, suggesting a potential offsetting or paradoxical effect when both conditions are present. In fact, one study indicated that both diabetes and underweight status are independent risk factors for TB incidence [12].

While previous studies have examined the influence of obesity and diabetes on TB prevalence and incidence, large-scale investigations of their association with treatment outcomes and mortality remain relatively scarce, particularly in East Asian populations. In this study, we analyzed data from two large TB cohorts in the Republic of Korea to examine the association of overweight (BMI ≥ 23 kg/m², per Asian criteria) with treatment outcomes and mortality during TB therapy, and whether these associations differ by diabetes status.

## Methods

### Multicenter prospective cohort of pulmonary TB

The cohort study of pulmonary TB (COSMOTB), a multicenter prospective observational cohort study, was conducted from 2019 to 2021 [13]. The main objective was to analyze various factors related to the anti-TB treatment outcomes. We recruited participants diagnosed with pulmonary TB, aged 19 years and older, from 18 university hospitals, and followed them from initiation of TB treatment to identify the treatment outcome. People with rifampicin-resistant TB were excluded from the current analysis.

### National TB registry database

The public-private mix project for TB care and prevention was expanded nationally across the Republic of Korea. This project involved surveillance of all the notified people receiving treatment for TB and monitoring various aspects until the completion of treatment. As part of this initiative, we established a prospective observational registry database, the Korean TB cohort (KTBC) [14]. We collected clinical and epidemiological information from patients at the time of TB diagnosis and upon the completion of their treatment. For this study, we enrolled people diagnosed with rifampicin-susceptible pulmonary TB from July 2018 to December 2020.

### Definition of Anti-TB treatment outcomes

Treatment outcomes were classified according to the Korean TB guidelines adopted from the World Health Organization. A favorable outcome was defined as treatment success, comprising all patients who were cured or completed treatment, based on clinical, microbiological, and radiological assessments recorded in the medical records. An unfavorable outcome was defined as a composite of death, treatment failure, loss to follow-up, still-on-treatment, and transfer-out. While the standard duration of first-line anti-TB [15] therapy is 6 months, treatment extension beyond this period is not uncommon in clinical practice due to adverse drug reactions, comorbid conditions, or delayed sputum/radiographic conversion. We defined still-on-treatment as cases in which treatment had not been completed by 365 days after initiation, and these cases were categorized as having an unfavorable outcome. The secondary outcome was all-cause mortality during treatment.

### Main exposure variable: overweight

The main exposure variable in this study was overweight. A Receiver Operating Characteristic (ROC) curve analysis was performed to assess the predictive power of body mass index (BMI) for unfavorable treatment outcomes. The ROC analysis yielded an area under the curve of 0.73 (95% confidence interval [CI]: 0.68–0.77, *p* < 0.001), with an optimal cutoff value of 23.7 kg/m² (Supplementary Fig. 1). Based on this result and the 2022 criteria of the Korean Society for the Study of Obesity [16], overweight was defined as BMI ≥ 23.0 kg/m². Participants were dichotomized into two groups: overweight (BMI ≥ 23.0 kg/m²) and non-overweight (normal and underweight; BMI < 23.0 kg/m²).

### Co-variables

Covariates included demographic and clinical factors known to influence TB treatment outcomes: sex, age, diabetes, malignancy, previous TB treatment history, initial TB-related symptoms, cavitation on chest radiography, and sputum acid-fast bacilli smear results. Diabetes was defined as having an HbA1c ≥ 6.5%, a random blood glucose ≥ 200 mg/dL, or a self-reported history of diabetes. Disease severity was categorized as mild (non-cavitary disease and negative smear result) or severe (cavitary disease or positive smear result) based on chest imaging and smear status.

### Statistical analysis

Continuous variables were presented as medians and interquartile ranges, while discrete variables were presented as frequencies and percentages. Baseline characteristics of patients were compared according to overweight, and univariable analyses were performed using the chi-square test for categorical variables. Regression analyses were conducted, and univariable analyses were performed to explore the association between treatment outcomes and overweight. Variables with a p-value of less than 0.20 in univariable logistic regression analyses were included in multivariable logistic regression analyses. Associations were estimated with multivariable logistic regression adjusting for demographic and clinical covariates. We report adjusted odds ratios (aORs) with 95% CIs and do not ascribe causal interpretation. We also stratified into key variables, such as age, sex, and diabetes, and conducted subgroup analysis to assess the association between treatment outcomes and overweight. Statistical significance was defined as a p-value of less than 0.05.

We further conducted prespecified subgroup analyses stratified by diabetes status to assess whether the association between BMI and TB treatment outcomes differed by diabetes status. Subgroup analyses were conducted to explore the associations between diabetes and BMI. For these analyses, participants were classified into six groups based on the presence or absence of diabetes and BMI categories. BMI categories were defined as follows: normal/underweight (BMI < 23.0 kg/m²), pre-obese (BMI 23.0 to < 25.0 kg/m²), and obese (BMI ≥ 25.0 kg/m²). Univariable and multivariable logistic regression analyses were performed to evaluate the associations between subgroup categories and the study outcomes. In all regression models, the reference group was participants without diabetes and with normal/underweight BMI. Analyses were conducted separately for the two primary endpoints: unfavorable treatment outcomes and all-cause mortality during TB treatment. The analysis was conducted using the R program.

### Sample size

We prespecified overweight (BMI ≥ 23.0 kg/m²) versus normal/underweight (BMI < 23.0 kg/m²) as the primary exposure contrast. For the primary endpoint (unfavorable treatment outcome), we estimated the minimum required sample size for a two-sided comparison of two independent proportions with unequal allocation (30% overweight vs. 70% normal/underweight BMI) [17], assuming an α of 0.05, a power of 80%, an overall event rate of 80% [13], and an absolute difference of 8% between groups (0.16 vs. 0.24). Using the normal approximation for two-proportion tests with an allocation ratio of 0.30:0.70, the required sample sizes are approximately 263 participants with overweight and 612 participants with normal/underweight BMI, for a total of 875 participants. Both cohorts exceed these thresholds, indicating adequate power for the primary analysis.

## Results

In the COSMOTB and KTBC databases, 1,055 and 18,433 participants were included in the final analysis, respectively (Supplemental Fig. 2). Approximately two thirds of participants were male and about half were aged 65 years and over (Table 1.). The proportions of overweight were 34.4% in the COSMOTB and 29.2% in the KTBC. In both databases, the prevalence of diabetes and the proportion of participants without initial TB-related symptoms were significantly higher among those with overweight.

**Table 1.** Baseline characteristics of enrolled individuals with pulmonary tuberculosis in a multicenter prospective cohort of pulmonary tuberculosis (COSMOTB) and a national tuberculosis registry database (Korea Tuberculosis Cohort, KTBC)

Variables	COSMOTB				KTBC			
	Total	Overweight	Normal/underweight	p-value	Total	Overweight	Normal/underweight	p-value
	(n = 1,055)	(n = 363)	(n = 692)		(n = 18,433)	(n = 5,387)	(n = 13,046)	
Male sex	687 (65.1)	241 (66.4)	446 (64.5)	0.575	1,1683 (63.4)	3,598 (66.8)	8,085 (62.0)	< 0.001
Age ≥ 65 years	466 (44.2)	164 (45.2)	302 (43.6)	0.680	9,085 (49.3)	2,629 (48.8)	6,456 (49.5)	0.398
Diabetes	324 (30.7)	127 (35.0)	197 (28.5)	0.035	4,111 (22.3)	1,489 (27.6)	2,622 (20.1)	< 0.001
Malignancy	98 (9.3)	31 (8.5)	67 (9.7)	0.620	1,805 (9.8)	503 (9.3)	1,302 (10.0)	0.182
Prior TB history	170 (16.1)	52 (14.3)	118 (17.1)	0.291	2,831 (15.4)	712 (13.2)	2,119 (16.2)	< 0.001
Extrapulmonary involvement	93 (8.8)	25 (6.9)	68 (9.8)	0.137	1,716 (9.3)	461 (8.6)	1,255 (9.6)	0.024
Presence of initial TB-related symptoms	707 (67.0)	217 (59.8)	490 (70.8)	< 0.001	11,774 (63.9)	3,187 (59.2)	8,587 (65.8)	< 0.001
Initial disease severity				0.110				< 0.001
Mild	626 (63.2)	227 (66.8)	399 (61.2)		12,248 (66.4)	3,915 (72.7)	8,333 (63.9)	
Severe	364 (36.8)	113 (33.2)	251 (38.5)		6,185 (33.6)	1,472 (27.3)	4,713 (36.1)	
Treatment outcome								
Unfavorable outcome	194 (18.4)	51 (14.0)	143 (20.7)	0.011	7,478 (40.6)	1,811 (33.6)	5,667 (43.5)	< 0.001
Mortality	58 (5.5)	12 (3.3)	46 (6.6)	0.034	2,127 (11.5)	407 (7.6)	1,720 (13.2)	< 0.001

*COSMOTB *Cohort Study of Pulmonary Tuberculosis, *KTBC* Korea Tuberculosis Cohort, *TB* Tuberculosis

Initial disease severity: Mild [smear (-) and cavity (-)] Severe [smear (+) or cavity (+)]

Overweight is defined as a body mass index of 23 kg/m^2^ or greater

In the COSMOTB database, the proportions of unfavorable outcome were 14.0% in the overweight group and 20.7% in the normal/underweight group (*p* = 0.011), and mortality proportions were 3.3% and 6.6%, respectively (*p* = 0.034). In the KTBC database, the proportions of unfavorable outcome were 33.6% and 43.4% (*p* < 0.001), and mortality proportions were 7.6% and 13.2% (*p* < 0.001), respectively. Detailed categories of treatment outcomes for the overall and overweight groups in both databases are provided in Supplementary Table 1.

**Fig. 1:**
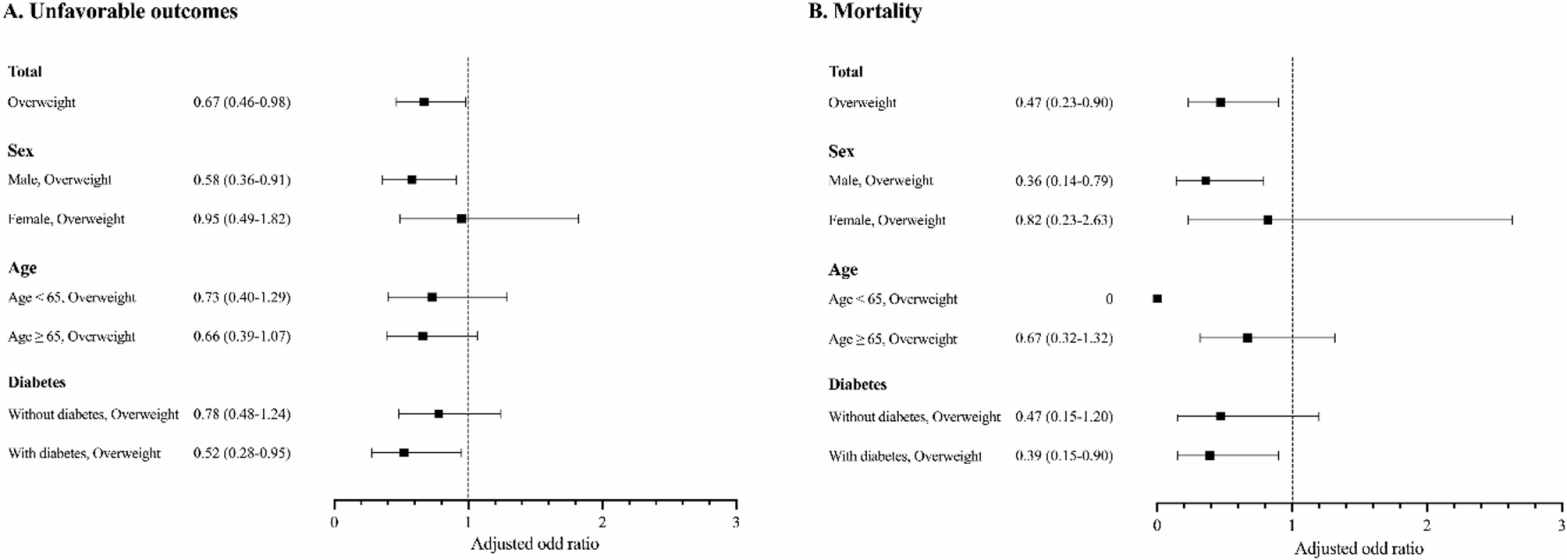
Adjusted odds ratios (aORs) for unfavorable treatment outcomes and all-cause mortality according to overweight status, stratified by sex, age, and diabetes status among patients with pulmonary tuberculosis in the multicenter prospective cohort (COSMOTB). Overweight was defined as a body mass index (BMI) ≥ 23 kg/m². Analyses were adjusted for age, sex, diabetes, malignancy, prior TB history, presence of TB-related symptoms, and initial disease severity. Error bars represent 95% confidence intervals (CIs). Variables with P-value < 0.20 in univariable analyses were included in the multivariable logistic regression model

In multivariable logistic regression, the aOR for overweight versus normal/underweight for unfavorable outcome was 0.67 (95% CI, 0.46–0.98) in COSMOTB and 0.67 (95% CI, 0.63–0.72) in KTBC (Table 2). Subgroup analyses by age, sex, and diabetes status showed that in the COSMOTB database, aORs for overweight versus normal/underweight were 0.58 (95% CI, 0.36–0.91) in males and 0.52 (95% CI, 0.28–0.95) in participants with diabetes (Fig. 1). The KTBC database showed similar subgroup estimates.

**Table 2:** Association between overweight and unfavorable treatment outcome among enrolled individuals with pulmonary tuberculosis: (A) a multicenter prospective cohort of pulmonary tuberculosis (COSMOTB) and (B) a National tuberculosis registry database (Korea tuberculosis cohort, KTBC)

Variables	Total	Favorable	Unfavorable	*P*-value	Adjusted OR (95% CI)
(A) COSMOTB					
Number of participants	1,055 (100)	861 (100)	194 (100)		
Overweight	363 (34.4)	312 (36.2)	51 (26.3)	0.011	0.67 (0.46–0.98)
Male sex	687 (65.1)	551 (64.0)	136 (70.1)	0.126	1.27 (0.87–1.85)
Age ≥ 65 years	466 (44.2)	350 (40.7)	116 (59.8)	< 0.001	2.06 (1.45–2.95)
Diabetes	324 (30.7)	245 (28.5)	79 (40.7)	0.001	1.40 (0.98–2.00)
Malignancy	98 (9.3)	65 (7.5)	33 (17.0)	< 0.001	2.71 (1.63–4.48)
Prior TB history	170 (16.1)	123 (14.3)	47 (24.2)	0.001	1.97 (1.28–2.99)
Extrapulmonary involvement	93 (8.8)	72 (8.4)	21 (10.8)	0.341	
Presence of TB-related symptoms	707 (67.0)	558 (64.8)	149 (76.8)	0.002	1.59 (1.07–2.41)
Initial severe disease	364 (36.8)	274 (33.8)	90 (50.3)	< 0.001	1.94 (1.36–2.76)

(B) KTBC					
Number of participants	18,433 (100)	13,051 (100)	5,382 (100)		
Overweight	5,387 (29.2)	4,198 (32.2)	1,189 (22.1)	< 0.001	0.67 (0.63–0.72)
Male sex	1,1683 (63.4)	8,166 (62.6)	3,517 (65.3)	< 0.001	1.19 (1.11–1.27)
Age ≥ 65 years	9,085 (49.3)	5,680 (43.5)	3,405 (63.3)	< 0.001	1.62 (1.52–1.72)
Diabetes	4,111 (22.3)	2,701 (20.7)	1,410 (26.2)	< 0.001	1.22 (1.13–1.31)
Malignancy	1,805 (9.8)	1,056 (8.1)	749 (13.9)	0.015	1.34 (1.21–1.49)
Prior TB history	2,831 (15.4)	1,930 (14.8)	901 (16.7)	0.001	1.29 (1.18–1.40)
Extrapulmonary involvement	1,716 (9.3)	1,234 (9.5)	482 (9.0)	0.289	
Presence of TB-related symptoms	11,774 (63.9)	8,045 (61.6)	3,729 (69.3)	< 0.001	1.34 (1.25–1.43)
Initial severe disease	6,185 (33.6)	4,176 (32.0)	2,009 (37.3)	< 0.001	1.22 (1.13–1.31)

*OR* Odds ratio, *CI* Confidence interval, *TB* Tuberculosis, *KTBC* Korean Tuberculosis Cohort, *COSMOTB* Cohort Study of Pulmonary Tuberculosis

Overweight is defined as a body mass index of 23 kg/m^2^ or greater

Reference category for “Presence of TB-related symptoms” is the absence of TB-related symptoms

Variables with P value < 0.20 in the univariable analyses were selected for the multivariable logistic regression analysis

**Fig. 2:**
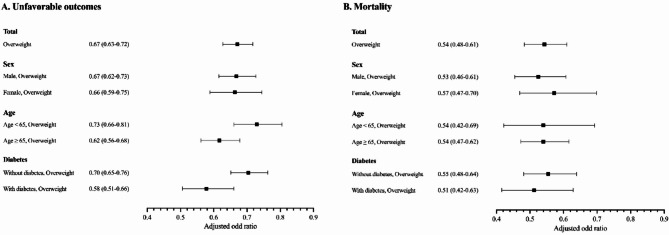
Adjusted odds ratios (aORs) for unfavorable treatment outcomes and all-cause mortality according to overweight status, stratified by sex, age, and diabetes status among patients with pulmonary tuberculosis in the national tuberculosis registry database (KTBC). Overweight was defined as a body mass index (BMI) ≥ 23 kg/m². Analyses were adjusted for age, sex, diabetes, malignancy, prior TB history, presence of TB-related symptoms, and initial disease severity. Error bars represent 95% confidence intervals (CIs). Variables with P-value < 0.20 in univariable analyses were included in the multivariable logistic regression model

Associations between overweight and all-cause mortality during treatment were observed in both COSMOTB (aOR, 0.49; 95% CI, 0.24–0.93) and KTBC (aOR, 0.54; 95% CI, 0.48–0.61) **(**Table 3). In subgroup analyses, aORs for overweight were 0.36 (95% CI, 0.14–0.79) in males and 0.39 (95% CI, 0.15–0.90) in participants with diabetes in the COSMOTB database, with similar estimates in the KTBC database (Fig. 2).

**Table 3:** Association between overweight and all-cause mortality among enrolled individuals with pulmonary tuberculosis: (A) a multicenter prospective cohort of pulmonary tuberculosis (COSMOTB) and (B) a National tuberculosis registry database (Korea tuberculosis cohort, KTBC)

Variables	Total	Survival	Mortality	*P*-value	Adjusted OR (95% CI)
(A) COSMOTB					
Number of participants	1,055 (100)	997 (100)	58 (100)		
Overweight	363 (34.4)	351 (35.2)	12 (20.7)	0.038	0.49 (0.24–0.93)
Male sex	687 (65.1)	643 (64.5)	44 (75.9)	0.097	1.73 (0.92–3.42)
Age ≥ 65 years	466 (44.2)	420 (42.1)	46 (79.3)	< 0.001	4.89 (2.51–10.31)
Diabetes	324 (30.7)	292 (29.3)	32 (55.2)	0.002	2.52 (1.43–4.5)
Malignancy	98 (9.3)	84 (8.4)	14 (24.1)	0.032	2.18 (1.04–4.35)
Prior TB history	170 (16.1)	159 (15.9)	11 (19.0)	0.672	
Extrapulmonary involvement	93 (8.8)	85 (8.5)	8 (13.8)	0.255	
Presence of TB-related symptoms	707 (67.0)	662 (66.4)	45 (77.6)	0.307	1.44 (0.74–2.99)
Initial severe disease	364 (36.8)	662 (66.4)	45 (77.6)	0.274	1.39 (0.77–2.49)

(B) KTBC					
Number of participants	18,433 (100)	16,306 (100)	2,127 (100)		
Overweight	5,387 (29.2)	4,980 (30.5)	407 (19.1)	< 0.001	0.54 (0.48–0.61)
Male sex	1,1683 (63.4)	10,266 (63.0)	1,417 (66.6)	0.001	1.31 (1.18–1.45)
Age ≥ 65 years	9,085 (49.3)	7,404 (45.4)	1,681 (79.0)	< 0.001	4.21 (3.76–4.71)
Diabetes	4,111 (22.3)	3,507 (21.5)	604 (28.4)	< 0.001	1.23 (1.10–1.36)
Malignancy	1,805 (9.8)	1,361 (8.3)	444 (20.9)	< 0.001	2.52 (2.21–2.86)
Prior TB history	2,831 (15.4)	2,493 (15.3)	338 (15.9)	0.469	
Extrapulmonary involvement	1,716 (9.3)	1,520 (9.3)	196 (9.2)	0.873	
Presence of TB-related symptoms	11,774 (63.9)	10,178 (62.4)	1,596 (75.0)	< 0.001	1.66 (1.48–1.85)
Initial severe disease	6,185 (33.6)	5,305 (32.5)	880 (41.4)	< 0.001	1.37 (1.24–1.51)

*OR* Odds ratio, *CI* Confidence interval, *TB* Tuberculosis, *KTBC* Korean Tuberculosis Cohort, *COSMOTB* Cohort Study of Pulmonary Tuberculosis

Overweight is defined as a body mass index of 23 kg/m^2^ or greater

Reference category for “Presence of TB-related symptoms” is the absence of TB-related symptoms

Variables with P value < 0.20 in the univariable analyses were selected for the multivariable logistic regression analysis

**Table 4:** Stratum-specific adjusted odds ratios of unfavorable outcome and mortality and effect of interaction between categories of body mass index and diabetes: (A) a multicenter prospective cohort of pulmonary tuberculosis (COSMOTB) and (B) a National tuberculosis registry database (Korea tuberculosis cohort, KTBC)

(A) COSMOTB		
Outcome	No diabetes	Diabetes
Unfavorable outcome		
Normal and underweight	1.00	1.61 (1.05–2.44)
Pre-obese	0.89 (0.49–1.56)	0.97 (0.47–1.88)
Obesity	0.61 (0.29–1.18)	0.61 (0.22–1.41)
Mortality		
Normal and underweight	1.00	2.72 (1.43–5.24)
Pre-obese	0.57 (0.13–1.75)	1.39 (0.44–3.69)
Obesity	0.43 (0.07–1.53)	0.95 (0.15–3.49)

(B) KTBC		
	No diabetes	Diabetes
Unfavorable outcome		
Normal and underweight	1.00	1.29 (1.18–1.41)
Pre-obese	0.75 (0.68–0.82)	0.79 (0.68–0.91)
Obesity	0.64 (0.57–0.72)	0.72 (0.62–0.85)
Mortality		
Normal and underweight	1.00	1.25 (1.10–1.41)
Pre-obese	0.55 (0.47–0.66)	0.64 (0.50–0.82)
Obesity	0.55 (0.45–0.68)	0.65 (0.49–0.86)

*TB* Tuberculosis, *KTBC* Korean Tuberculosis Cohort, *COSMOTB* Cohort Study of Pulmonary Tuberculosis

Body mass index (BMI) was categorized into four groups; (1) obese, greater than or equal to 25 kg/m^2^, (2) pre-obese, 23–24.9 kg/m^2^; (3) normal, 18.5–22.9 kg/m^2^, and (4) underweight, less than 18.5 kg/m^2^

Variables with P value < 0.20 in the univariable analyses were selected for the multivariable logistic regression analysis. The results were expressed as adjusted odds ratio with 95% confidence interval

In the combined BMI–diabetes subgroup analysis, using normal/underweight without diabetes as the reference, COSMOTB data showed that normal/underweight with diabetes had an aOR of 1.61 (95% CI, 1.05–2.44) for unfavorable outcomes and 2.72 (95% CI, 1.43–5.24) for mortality (Table 4A). Pre-obese and obese with diabetes had aORs below 1.0 for both outcomes, but these were not statistically significant.

In the KTBC database, normal/underweight with diabetes had an aOR of 1.29 (95% CI, 1.18–1.41) for unfavorable outcomes and 1.25 (95% CI, 1.10–1.41) for mortality (Table 4B). Pre-obese with diabetes had an aOR of 0.79 (95% CI, 0.68–0.91) and obese with diabetes had an aOR of 0.72 (95% CI, 0.62–0.85) for unfavorable outcomes. For mortality, the aORs were 0.81 (95% CI, 0.67–0.97) in pre-obese with diabetes and 0.80 (95% CI, 0.66–0.96) in obese with diabetes.

## Discussion

In this study, we examined the association between overweight and anti-TB treatment outcomes using data from a large multicenter prospective cohort, with validation in a nationwide TB registry. Given the observational nature of the analysis, our findings represent associations rather than direct causal effects and should be interpreted with caution. We found that overweight was significantly associated with a reduced risk of both unfavorable outcomes and mortality during TB treatment. This association persisted in key subgroups, including males and people with diabetes, suggesting that body mass index may be an important factor to consider in risk stratification and treatment planning for patients with TB.

Our previous analysis suggested a beneficial association of overweight with mortality during anti-TB treatment [17], and the current findings align with this previous study. We further demonstrated reduced risk of unfavorable outcome, including loss to follow-up and treatment failure. The association between overweight and better treatment outcomes may be explained by several factors. Previous studies have shown that low BMI is a strong predictor of poor treatment outcomes [18, 19], possibly due to the interplay between malnutrition, immune deficiency, and the severity of disease. However, mechanisms of beneficial effect of overweight were not fully investigated. Overweight could mitigate the effects of undernutrition on the metabolic and inflammatory dysregulations in people with TB. Overweight individuals may have greater nutritional reserves, which can support immune function and enhance resilience during anti-TB treatment. For example, the side effects of anti-TB drugs, one of the main reasons for ceasing treatment, could be reduced in the overweight individuals.

Diabetes is another important factor associated with unfavorable outcome during anti-TB treatment [13]. Our study results revealed that the association between overweight and a lower risk of unfavorable outcome was more pronounced in people with diabetes. Subgroup analyses further revealed that normal weight and underweight individuals with diabetes were at significantly higher risk for unfavorable outcomes and mortality, compared to those without diabetes. However, the deleterious effects of diabetes with TB treatment outcome appeared attenuated in the pre-obese and obese individuals. These results underline the hypothesis that adequate nutritional reserves and the metabolic advantages of higher BMI can counteract some of the physiological challenges posed by TB and diabetes. Understanding the complex interplay between BMI, comorbidities, and TB outcomes will be critical for optimizing patient care and improving long-term survival.

Several non–mutually exclusive explanations may account for the lower odds observed among participants with diabetes who had higher BMI. Greater energy reserves could mitigate catabolic stress during TB therapy, and adipose-derived signaling and inflammatory tone may differ across BMI strata. Alternatively, the association may reflect residual confounding or reverse causation. Individuals with higher BMI may have greater contact with the health system (e.g., routine care for metabolic comorbidities) and be diagnosed and treated earlier, may experience fewer catabolic sequelae (e.g., less TB-related weight loss), and may be more likely to receive specific diabetes therapies (e.g., metformin [20, 21], statins [22, 23]) that have been associated with improved TB outcomes in observational studies. Given these uncertainties, our findings should be interpreted as associations rather than causal effects.

The beneficial effect of being overweight was not consistent across all subgroups in our analysis, indicating that the relationship between BMI and anti-TB treatment outcomes is influenced by various patient-specific factors. For instance, in male participants, being overweight was associated with lower odds ratios for both unfavorable outcomes and mortality. This suggests that overweight status may provide a distinct physiological or metabolic advantage in male TB patients, potentially linked to differences in fat distribution, hormonal factors, or immune responses between the sexes. The findings suggest that BMI should be considered alongside other demographic and clinical factors when developing individualized treatment plans for TB patients.

There are several limitations to this study. First, given its observational design, the findings should be interpreted as associations rather than causal effects. Although we adjusted for key clinical covariates in prespecified models, residual confounding cannot be entirely excluded, and causality cannot be definitively established. We lacked direct measures of socioeconomic status, dietary factors or nutritional supplementation, detailed glycemic control (e.g., longitudinal HbA1c), medication classes, and physical activity, all of which may influence both BMI and TB outcomes. Future studies with patient-level data on socioeconomic position, nutrition, diabetes phenotypes, and pharmacotherapy are needed to assess generalizability and clarify potential mechanisms. Second, the impact of diabetes on TB outcomes may vary according to glycemic control, presence of complications, and the use of specific antidiabetic or lipid-lowering agents [24–27]. For example, metformin [20, 21] has been associated with reduced TB mortality and enhanced host immune responses, while statins [22, 23], owing to their anti-inflammatory and immunomodulatory properties, have also been linked to lower TB incidence in observational studies. However, medication-specific data were not available in our cohort, precluding assessment of their potential modifying effects. Future studies should investigate the role of these agents as part of host-directed strategies to optimize TB treatment outcomes in patients with diabetes. Third, the BMI cutoff applied in our analysis (≥ 23 kg/m² for overweight) was derived from ROC analysis and aligned with the Korean Society for the Study of Obesity criteria. However, this cutoff may not be directly generalizable to other ethnic groups or populations, as the optimal BMI threshold for predicting TB outcomes may vary by age, sex, and ethnicity. Further validation in diverse cohorts is warranted. Fourth, our BMI exposure was categorized (per Asian thresholds) to facilitate clinical interpretability, however, categorization entails information loss and may obscure non-linear associations with TB outcomes. We did not model BMI as a continuous variable and therefore we cannot exclude U- or J-shaped relationships, particularly at the lower and upper tails of the distribution. In addition, very high BMI (“super-obesity,” ≥30 or ≥ 35 kg/m²) was uncommon in our cohorts, yielding imprecise estimates and limiting evaluation of these strata. The reference category combined normal weight and underweight, which may attenuate or exaggerate contrasts at the low end and obscure potential U- or J-shaped relationships. Future studies in settings with a wider BMI range should prespecify continuous BMI modeling with flexible terms, separate underweight from normal weight, and incorporate central adiposity/body-composition measures (e.g., waist circumference) as well as longitudinal weight change during treatment to characterize potential non-linearity more precisely and to improve generalizability.

Fourth, the BMI categorization used in this study was not designed to assess potential non-linear (U- or J-shaped) relationships between BMI and TB outcomes. In addition, super-obesity (BMI > 30 or > 35 kg/m²) was extremely rare in our study population, limiting statistical power to evaluate these subgroups separately. Consequently, our analysis cannot exclude the possibility of a U- or J-shaped association, and future studies with larger and more diverse populations are needed to explore these non-linear relationships in greater detail.

## Conclusion

While underweight is universally recognized as a risk factor for poor outcomes, our current findings suggest that overweight could be associated with better outcomes, particularly in the context of comorbidities like diabetes. However, this association should be interpreted with caution given the observational design and the absence of data on diabetes severity, glycemic control, and treatment details. These findings highlight the potential value of incorporating BMI into TB risk stratification models and of tailoring interventions to address the diverse nutritional needs of TB patients. Further research, ideally incorporating detailed diabetes-related variables and conducted in diverse populations, is warranted to elucidate the mechanisms underlying the observed associations and to assess their generalizability to other TB treatment settings.

## Supplementary Information

Supplementary Material 1.

## Data Availability

The ownership of the primary datasets lies with the Korea Disease Control and Prevention Agency (KDCA) and the Korea National Institute of Health (KNIH). The de-identified datasets generated and/or analysed during the current study can be made available for replication purposes upon reasonable request. Interested researchers should first contact the corresponding author, who will provide detailed instructions for submitting a request to the KDCA/KNIH, including required documents such as a brief study proposal, institutional review board (IRB) approval, and a data use agreement. Access will be granted only with permission from the KDCA/KNIH and will be limited to the scope of the approved research.

## Abbreviations

TB: Tuberculosis
BMI: Body Mass Index
COSMOTB: Cohort Study Of Pulmonary Tuberculosis
KTBC: Korean Tuberculosis Cohort
ROC: Receiver Operating Characteristic
CI: Confidence Interval
aOR: Adjusted Odds Ratio
