# Supplementary material for: Association of overweight with treatment outcomes in pulmonary tuberculosis
Source: BMC Infect Dis. 2025 Oct 8;25:1250. doi: 10.1186/s12879-025-11669-w (PMC12506272; doi:10.1186/s12879-025-11669-w)
Supplement: Supplementary file 1 — Supplementary Material 1. [file 12879_2025_11669_MOESM1_ESM.docx]

**Supplementary**

**Supplemental Figure 1.** Receiver operating characteristic curve analysis of body mass index as a predictor of unfavorable outcome among individuals with pulmonary tuberculosis


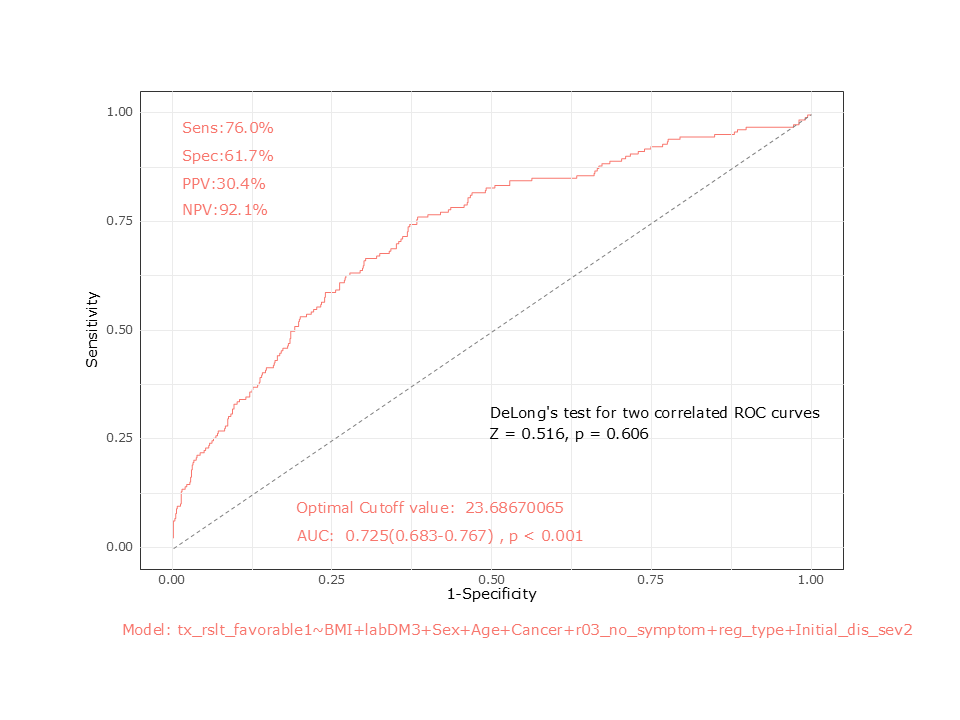


**Supplemental Figure 2.** Study flow chart presenting participants with pulmonary tuberculosis in the national Korea Tuberculosis Cohort (KTBC) registry database and the multicenter prospective cohort study of pulmonary tuberculosis (COSMOTB)

KTBC, Korean tuberculosis cohort; COSMOTB, cohort study of pulmonary tuberculosis; TB, tuberculosis; RIF, rifampicin

**Supplementary Table 1.** Detailed categories of tuberculosis treatment outcomes for the overall and overweight groups in the COSMOTB and KTBC datasets.

(A)

| Outcome category | COSMOTB | | | | | |
| --- | --- | --- | --- | --- | --- | --- |
|  | Total | | Overweight | | Normal/Underweight | |
|  | (n = 1055) | | (n = 363) | | (n = 692) | |
|  | n | % | n | % | n | % |
| Treatment success | 861 | 81.6 | 312 | 86.0 | 549 | 79.3 |
| Treatment failed | 4 | 0.4 | 3 | 0.8 | 1 | 0.1 |
| Died | 14 | 1.3 | 4 | 1.1 | 10 | 1.4 |
| Lost to follow-up | 57 | 5.4 | 12 | 3.3 | 45 | 6.5 |
| Not evaluated | 57 | 5.4 | 13 | 3.6 | 44 | 6.4 |
| Still-on-treatment | 62 | 5.9 | 19 | 5.2 | 43 | 6.2 |

(B)

| Outcome category | KTBC | | | | | |
| --- | --- | --- | --- | --- | --- | --- |
|  | Total | | Overweight | | Normal/Underweight | |
|  | (n = 18,433) | | (n = 5,387) | | (n = 13,046) | |
|  | n | % | n | % | n | % |
| Treatment success | 10,955 | 59.4 | 3,576 | 66.4 | 7,379 | 56.6 |
| Treatment failed | 23 | 0.1 | 6 | 0.1 | 17 | 0.1 |
| Died | 2,127 | 11.5 | 407 | 7.6 | 1,720 | 13.2 |
| Lost to follow-up | 361 | 2.0 | 99 | 1.8 | 262 | 2.0 |
| Not evaluated | 2,871 | 15.6 | 677 | 12.6 | 2,194 | 16.8 |
| Still-on-treatment | 2,096 | 11.4 | 622 | 11.5 | 1,474 | 11.3 |

KTBC, Korean tuberculosis cohort; COSMOTB, cohort study of pulmonary tuberculosis.

Values are presented as number (percentage). Overweight was defined as BMI ≥23.0 kg/m²; normal/underweight as BMI <23.0 kg/m². Treatment outcomes were classified according to the Korean tuberculosis guidelines adopted from the World Health Organization.
